# Cytological and transcriptomic analysis to unveil the mechanism of web blotch resistance in Peanut

**DOI:** 10.1186/s12870-023-04545-9

**Published:** 2023-10-26

**Authors:** Xiaohui Wu, Ziqi Sun, Feiyan Qi, Hua Liu, Mingbo Zhao, Juan Wang, Mengmeng Wang, Ruifang Zhao, Yue Wu, Wenzhao Dong, Zheng Zheng, Xinyou Zhang

**Affiliations:** 1 The Shennong Laboratory, Institute of Crop Molecular Breeding, Key Laboratory of Oil Crops in Huang-Huai-Hai Plains, Ministry of Agriculture/Henan Provincial Key Laboratory for Oil Crop Improvement, Henan Academy of Agricultural Sciences, Zhengzhou, Henan 450002 China; 2 College of Agronomy, Henan Agricultural University, Zhengzhou, Henan 450046 China

**Keywords:** Peanut, Web blotch, Cytological examination, RNA-seq, Cysteine

## Abstract

**Background:**

Peanut is an important oil crop worldwide. Peanut web blotch is a fungal disease that often occurs at the same time as other leaf spot diseases, resulting in substantial leaf drop, which seriously affects the peanut yield and quality. However, the molecular mechanism underlying peanut resistance to web blotch is unknown.

**Results:**

The cytological examination revealed no differences in the conidium germination rate between the web blotch-resistant variety ZH and the web blotch-susceptible variety PI at 12–48 hpi. The appressorium formation rate was significantly higher for PI than for ZH at 24 hpi. The papilla formation rate at 36 hpi and the hypersensitive response rate at 60 and 84 hpi were significantly higher for ZH than for PI. We also compared the transcriptional profiles of web blotch-infected ZH and PI plants at 0, 12, 24, 36, 48, 60, and 84 hpi using an RNA-seq technique. There were more differentially expressed genes (DEGs) in ZH and PI at 12, 36, 60, and 84 hpi than at 24 and 48 hpi. Moreover, there were more DEGs in PI than in ZH at each time-point. The analysis of metabolic pathways indicated that pantothenate and CoA biosynthesis; monobactam biosynthesis; cutin, suberine and wax biosynthesis; and ether lipid metabolism are specific to the active defense of ZH against YY187, whereas porphyrin metabolism as well as taurine and hypotaurine metabolism are pathways specifically involved in the passive defense of ZH against YY187. In the protein-protein interaction (PPI) network, most of the interacting proteins were serine acetyltransferases and cysteine synthases, which are involved in the cysteine synthesis pathway. The qRT-PCR data confirmed the reliability of the transcriptome analysis.

**Conclusion:**

On the basis of the PPI network for the significantly enriched genes in the pathways which were specifically enriched at different time points in ZH, we hypothesize that serine acetyltransferases and cysteine synthases are crucial for the cysteine-related resistance of peanut to web blotch. The study results provide reference material for future research on the mechanism mediating peanut web blotch resistance.

**Supplementary Information:**

The online version contains supplementary material available at 10.1186/s12870-023-04545-9.

## Background

Peanut is an important oil crop, accounting for approximately 50% of the total oil crop output in China [1]. It is also an important source of protein, dietary minerals, and vitamins as well as edible oil [2]. Web blotch is a foliar disease that adversely affects peanut production. It was firstly detected in peanut-producing province Shandong and Liaoning in 1982 [3]. With the recent changes in climatic and environmental conditions, the continuous cropping of peanut, and the expansion of the peanut-planting area, peanut web blotch has become one of the most serious leaf diseases affecting peanut production in China.

Web blotch is caused by the imperfect fungus *Didymella arachidicola* (also known as *Phoma arachidicola* or *Peyronella arachidicola*) [4, 5]. A *D. arachidicola* infection affects the photosynthetic efficiency of plants, leading to early leaf shedding, thereby affecting peanut quality and decreasing the peanut yield by 10–20% or more than 50% in severe cases [6]. Breeding web blotch-resistant cultivars is considered to be the most economical and environmentally friendly approach to avoid peanut production loss [7, 8]. Moreover, clarifying the genetic basis of disease resistance will enable researchers and breeders to develop resistant peanut cultivars against web blotch, thereby ensuring food safety without damaging the environment [9].

Pathogen-associated molecular pattern (PAMP)-triggered immunity (PTI) and effector-triggered immunity (ETI) are the two kinds of innate immune systems for plants to sense and limit the growth of pathogens. PTI is mediated by pattern recognition receptors on the cell surface that sense PAMPs. The intracellular changes related to PTI include the rapid transport of ions through the plasma membrane as well as the activation of MAP kinase, production of reactive oxygen species (ROS), expression of disease resistance genes, accumulation of callose, and strengthening of the cell wall [10, 11]. In addition, PAMPs can also induce the formation of papillae, which function as a structural barrier. More specifically, the convex envelope of the fungal invasion structure forms at the invasion site on the inner side of the plant cell wall, were degradative enzymes and callose accumulate to prevent pathogen infections [10]. Studies on papillae in barley infected with powdery mildew revealed that MLO negatively regulates the formation of papillae; a mutation to the MLO-encoding gene leads to resistance, indicative of the importance of papillae for plant defenses against fungal invasions [12, 13]. ETI involves the recognition of an effector secreted by an R protein comprising a nucleotide-binding site and a leucine-rich repeat [14]. Additionally, ETI is often accompanied by localized cell death (i.e., hypersensitive response; HR), which makes it difficult for pathogens to obtain the nutrients needed for growth and colonization, ultimately inhibiting the dispersal of pathogens and leading to disease resistance [14, 15]. The production of ROS is one of the earliest defense responses to a pathogen infection. In the early infection stage, the formation of ROS associated with PTI is weakly induced. In contrast, the production of ROS related to ETI is more intense and occurs over a longer period. It is also accompanied by the accumulation of nitric oxide (NO) and salicylic acid (SA), which promote hypersensitivity along with ROS [16]. Therefore, HR is typically associated with ETI and helps to activate the defense of neighboring cells as well as systemic acquired resistance, which is an SA-mediated broad-spectrum form of disease resistance involving the systematic activation of some defense responses [17].

Recent transcriptomics-based research has elucidated the mechanism underlying host–pathogen interactions and identified resistance-related genes and metabolites in plants, resulting in substantial progress in plant disease research and the development of new methods for breeding disease-resistant plants [18]. To date, 10 candidate effectors that may play important roles in plant interactions with *Fusarium oxysporum* have been identified using transcriptomics techniques [19]. In an earlier study on the interaction between rice and the fungus responsible for rice blast, 58 candidate effectors were screened [20]. Many genes related to disease resistance have been identified in wheat [21], maize [22], cucumber [23], *Gastrodia* [24], *Gerbera* [25], and other species. Furthermore, key metabolic pathways in plant responses to pathogen infections have been determined, providing the theoretical basis for research on plant disease resistance mechanisms [26]. Nevertheless, no detailed analysis has been performed to elucidate the molecular regulatory mechanisms underlying the response of peanut to *D. arachidicola* infection.

In this study, we compared the *D. arachidicola* infection process of leaves of different peanut resistant varieties by microscope, and analyzed the time-resolved transcription of them after infection by *D. arachidicola* by RNA-Seq technology. The significant enrichment pathway and differential expression gene (DEG) related to pathogen resistance were analyzed and explored, and several potential candidate genes involved in resistance to *D. arachidicola* infection were identified. These results provide a new understanding of the molecular mechanism of peanut resistance to *D. arachidicola* infection, and will promote the cultivation of peanut varieties with lasting and web blotch disease resistance in the future.

## Results

### Web blotch disease progression in Peanut plants inoculated with YY187

**Fig. 1:**
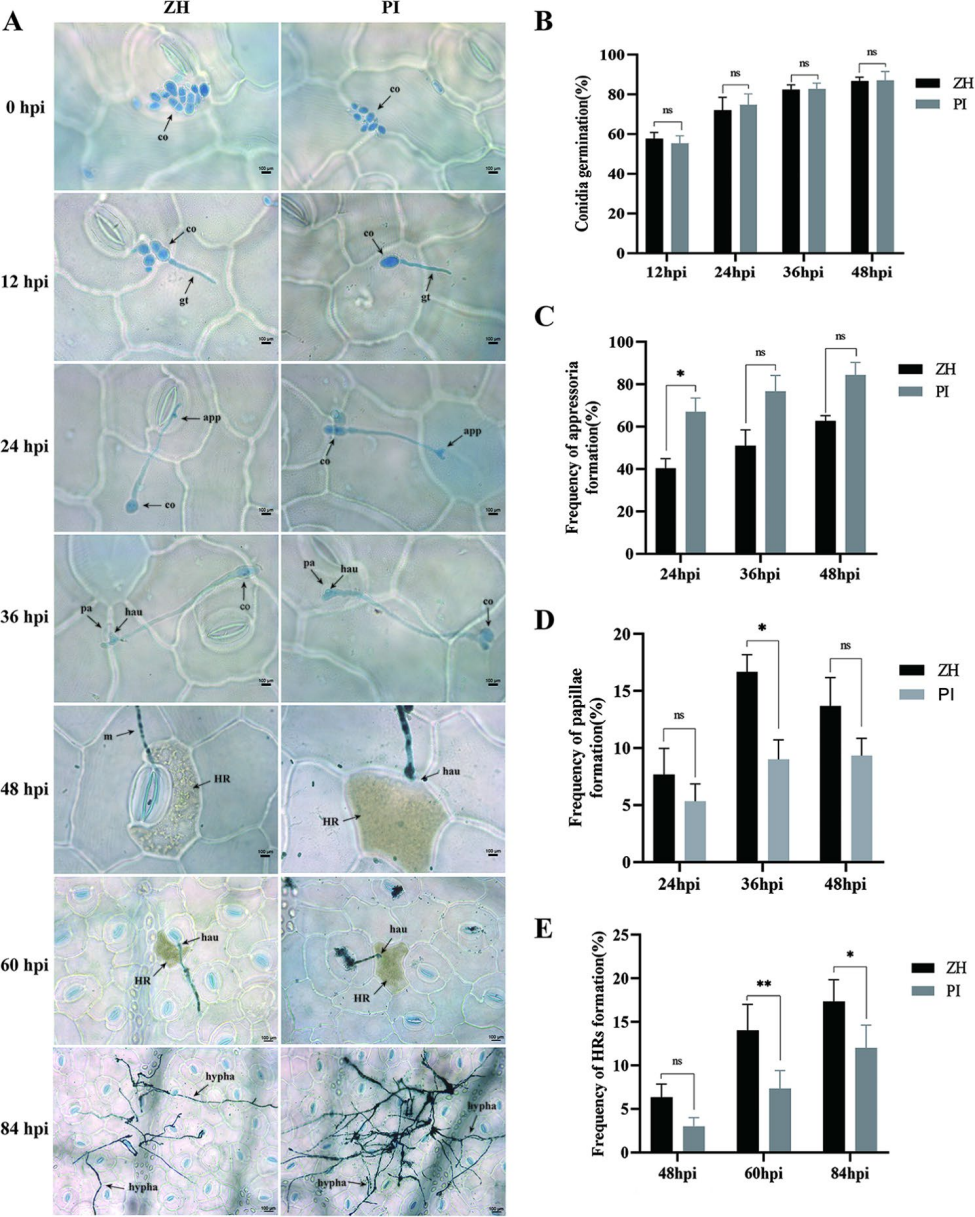
Web blotch disease progression in ZH and PI leaves infected with YY187. The leaves were stained with trypan blue and DAB prior to the microscopic examination. (**A**) Defense responses and pathogen infection structures for four ZH and PI samples at 0, 24, 36, 48, 60, and 84 hpi. Co: Conidia; gt: germ tube; app: appressorium; hau: haustorium; p.a.: papillae; HR: hypersensitive response. The scale bar represents 100 μm. (**B**) Conidium germination rates for ZH and PI at 12, 24, 36, and 48 hpi. Each column represents the average germination rate of 300 conidia. (**C**) Appressorium formation rates for ZH and PI at 24, 36, and 48 hpi. Each column represents the average appressorium formation rate of 300 conidia. (**D**) Papilla formation rates for ZH and PI at 24, 36, and 48 hpi. Each column represents the average papilla formation rate at 150 infection sites, which were calculated using three replicates. (**E**) HR rates for ZH and PI at 48, 60, and 84 hpi. Each column represents the average HR rate at 150 infection sites. All experiments were completed using three biological replicates, each of which included two leaflets from three plants. Ns represents no significant difference, whereas asterisks indicate significant differences as determined by a one-way ANOVA (*P < 0.05, **P < 0.01)

The severity of the YY187 infection differed significantly between the susceptible peanut variety PI343384 (PI) and the resistant peanut variety Zheng 8903 (ZH). Obvious chlorotic lesions were detected on the leaves of PI at 14 days post-inoculation, whereas the ZH leaves were symptomless (Fig. S1). The leaves collected at 0, 12, 24, 36, 48, 60, and 84 h post-inoculation (hpi) were stained with trypan blue and DAB and analyzed using a microscope (Fig. 1). There was no significant difference in the conidium germination rates between ZH (resistant variety) and PI (susceptible variety) at 12, 24, 36, and 48 hpi (Fig. 1A and B). At 24 hpi, the elongated end of the germ tube began to expand and form an appressorium, but the appressorium formation rate was significantly higher for PI than for ZH (Fig. 1A and C). A few papilla structures were detected at the haustorium penetration site of the ZH and PI plants at 24 hpi. At the later time-points (36 and 48 hpi), there was an increase in the formation of papilla structures in ZH and PI, but the papilla formation rate differed significantly between ZH and PI only at 36 hpi (Fig. 1A and D). Moreover, HR was first detected at 48 hpi and the HR of ZH was significantly more extensive than that of PI at 60 and 84 hpi (Fig. 1A and E).

### Transcriptomic changes in ZH and PI infected with YY187

On the basis of the microscopic examination, the samples collected at seven time-points (T0, T12, T24, T36, T48, T60, and T84) were used for transcriptome sequencing analysis by the Illumina HiSeq 2500 platform. For each sample, three biological replicates were conducted and a total of 42 cDNA libraries were constructed and sequenced. After filtering the raw data, 326.15 Gb clean data were retained (Table S1). The average Q20 and Q30 scores were 97.98% and 93.9%, respectively, and the average GC content was 44.56% (Table S1). The reads were mapped to the reference genome of peanut cultivar Tifrunner 2.0 (https://www.peanutbase.org/peanut_genome), with an average mapping rate of 98.26% (Table S1). The significant differences in the pearson correlation coefficients between samples (Fig. S2) and principal component analyses (PCA) (Fig. S3) reflected the differences between samples at T0 and other time-points (T12, T24, T36, T48, T60, and T84). Thus, the YY187 infection induced large changes in transcription levels in peanut.

### Identification of differentially expressed genes related to web blotch resistance

**Fig. 2:**
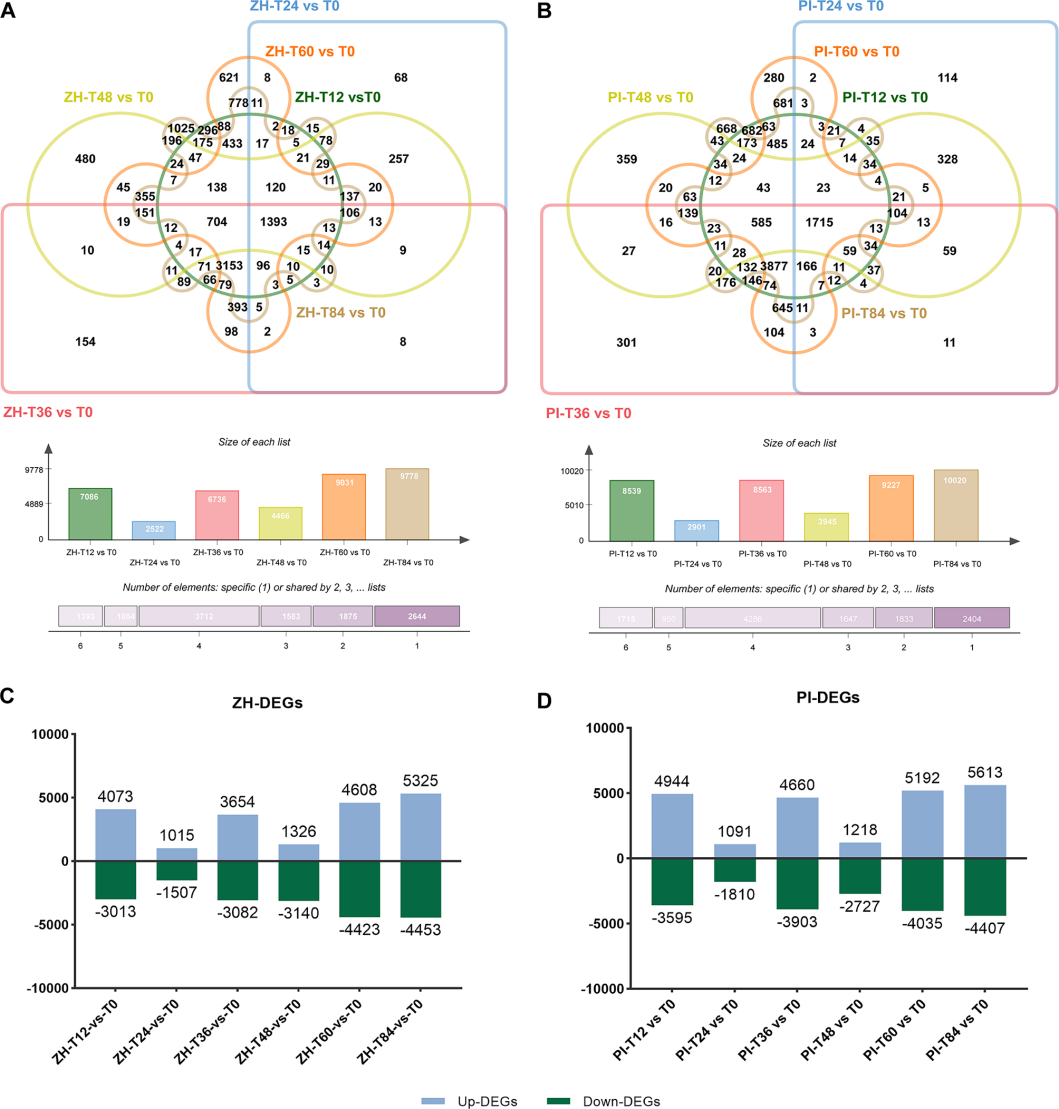
Presentation of DEGs in ZH and PI at different time-points post-inoculation. (**A**) Overlapping DEGs in ZH at T12, T24, T36, T48, T60, and T84. (**B**) Overlapping DEGs in PI at T12, T24, T36, T48, T60, and T84. (**C**) DEGs in ZH at T12, T24, T36, T48, T60, and T84 (compared with T0). (**D**) DEGs in PI at T12, T24, T36, T48, T60, and T84 (compared with T0)

For ZH, in response to the YY187 infection, 7,086, 2,522, 6,736, 4,466, 9,031, and 9,778 differentially expressed genes (DEGs) were detected at T12, T24, T36, T48, T60, and T84, respectively (Fig. 2A), of which 4,073, 1,015, 3,654, 1,326, 4,608, and 5,325 were up-regulated DEGs and 3,013, 1,507, 3,082, 3,140, 4,423, and 4,453 were down-regulated DEGs (Fig. 2C). After PI was infected with YY187, 8,539, 2,901, 8,563, 3,945, 9,227, and 10,020 DEGs were detected at T12, T24, T36, T48, T60, and T84, respectively (Fig. 2B), of which 4,944, 1,091, 4,660, 1,218, 5,192, and 5,613 were up-regulated DEGs and 3,595, 1,810, 3,903, 2,727, 4,035, and 4,407 were down-regulated DEGs (|log_2_(fold-change)|≥2 and FDR < 0.01) (Fig. 2D).

According to the gene expression analysis, the number of genes involved in the peanut response to disease varied among the pathogen infection stages. There were fewer DEGs at T12, T24, T36, T60, and T84 in ZH than in PI, implying the defense response of susceptive variety requires more induced genes than that of resistant variety after a pathogen infection. There were substantially more DEGs at T12, T36, T60, and T84 than at T24 and T48, indicating that T12, T36, T60 and T84 may be crucial for the interaction between the pathogen and peanut plants and for the resistance to web blotch.

To further investigate the specific genes affected at different disease developmental stages, we compared the DEGs in ZH and PI at six time-points. There were 296, 68, 154, 480, 621, and 1,025 unique DEGs in ZH (Fig. 2A) and 682, 114, 301, 359, 280, and 668 unique DEGs in PI at T12, T24, T36, T48, T60, and T84, respectively (Fig. 2B). There were also 1,393 and 1,715 shared DEGs in ZH and PI, respectively (Fig. 2A and B).

### Gene Ontology and Kyoto Encyclopedia of genes and genomes enrichment analyses of DEGs

The pathogen-induced DEGs in ZH and PI at different time-points were functionally annotated on the basis of Gene Ontology (GO) and Kyoto Encyclopedia of Genes and Genomes (KEGG) enrichment analyses. The GO enrichment analysis divided the DEGs in ZH and PI into three categories (molecular function, cellular component, and biological process) (Fig. S4). The main biological process GO terms assigned to the DEGs in ZH and PI at different time-points were cellular process (GO:0009987), metabolic process (GO:0008152), response to stimulation (GO:0050896), biological regulation (GO:0065007), localization (GO:0051179), and regulation of biological process (GO:0050789). The enriched molecular function GO terms included catalytic activity (GO:0003824), binding (GO:0005488), and transporter activity (GO:0005215), whereas the main cellular component GO terms were cellular anatomical entity (GO:0110165), protein-containing complex (GO:0032991), and virion component (GO:0044423) (Fig. S4).

**Fig. 3:**
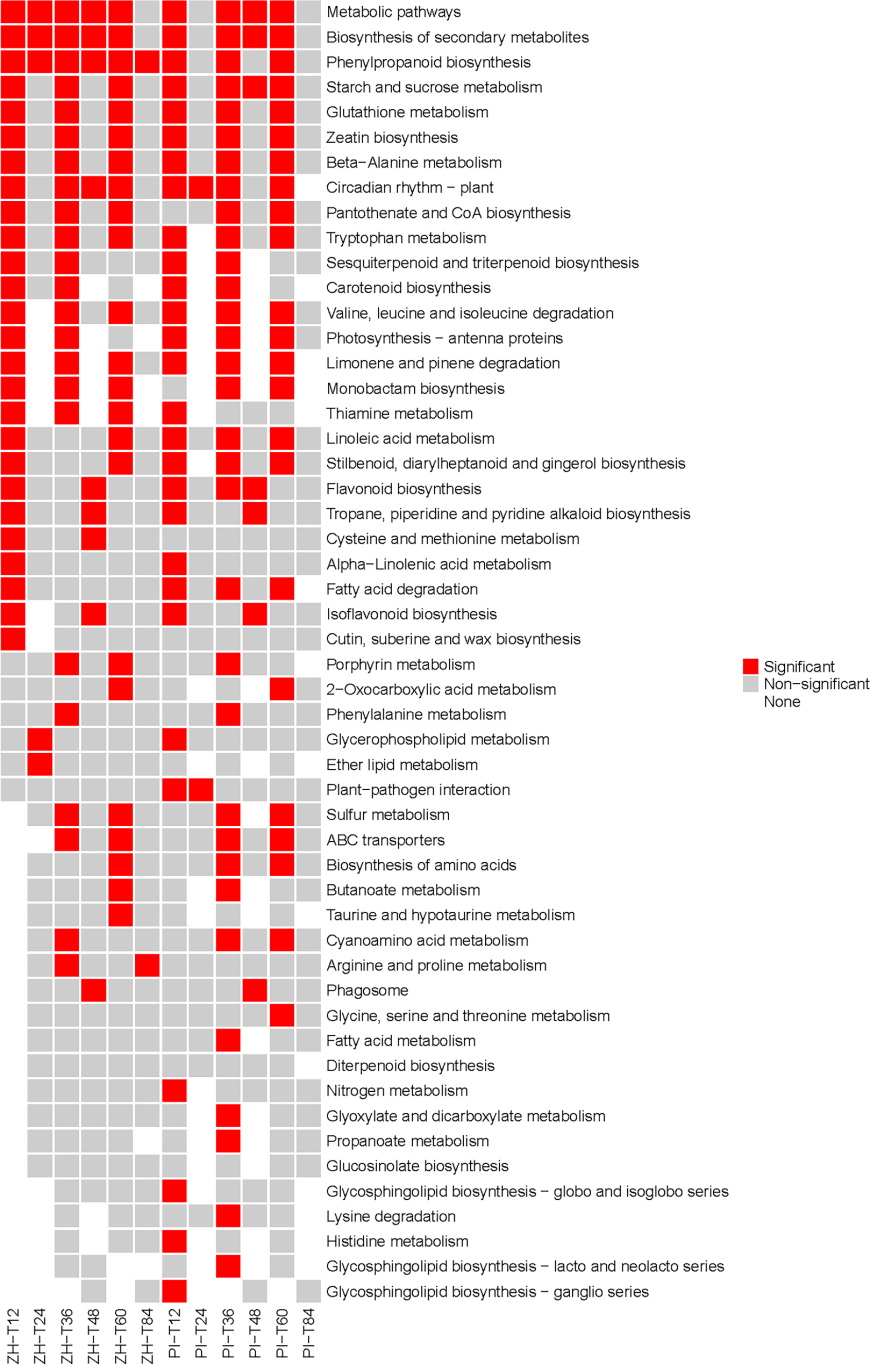
Significantly enriched KEGG pathways at T12, T24, T36, T48, T60, and T84 in ZH and PI. The KEGG image used in this figure is licensed by KEGG copyright and has been changed accordingly

The DEGs of ZH and PI at six time points were significantly enriched to 2–32 KEGG pathways (q < 0.05) (Fig. 3). Most of the pathways were significantly enriched at T12, T36, and T60, but a few pathways were significantly enriched at T24, T48, and T84. Only the DEGs of ZH and PI at T12, T36 and T60 were significantly enriched to glutathione metabolism; maize protein biosynthesis; β-alanine metabolism; tryptophan metabolism; valine, leucine and isoleucine degradation; and limonene and pinene degradation (Fig. 3). Phenylpropanoid biosynthesis was a significantly enriched pathway at all time-points in ZH, but only at T12, T36, and T60 in PI (Fig. 3). There were also some pathways that were significantly enriched at a specific time-point. For example, among the DEGs in ZH, cutin, suberine and wax biosynthesis was a significantly enriched pathway only at T12, whereas ether lipid metabolism was a significantly enriched pathway only at T24 and taurine and hypotaurine metabolism was a significantly enriched pathway only at T60. Arginine and proline metabolism was a significantly enriched pathway only at T36 and T84 (Fig. 3). In addition, pantothenate and CoA biosynthesis and monobactam biosynthesis were significantly enriched pathways at T12, T36, and T60 in ZH, but only at T36 and T60 in PI. Porphyrin metabolism was a significantly enriched pathway at T36 and T60 in ZH, but only at T36 in PI (Fig. 3). The above results indicated that T12, T36, and T60 may be critical time-points for peanut responses to web blotch. Furthermore, ZH may have evolved a series of molecular defense strategies to restrict the development of the pathogen causing web blotch. Pathways mediating cutin, suberine, and wax biosynthesis and ether lipid metabolism may be involved in the first line of defense, whereas taurine and hypotaurine metabolism may be induced later during the peanut defense response. These pathways combined to inhibit the development of web blotch in peanut.

### DEGs associated with germination and invasion inhibition during the early stage of the YY187 Infection

**Fig. 4:**
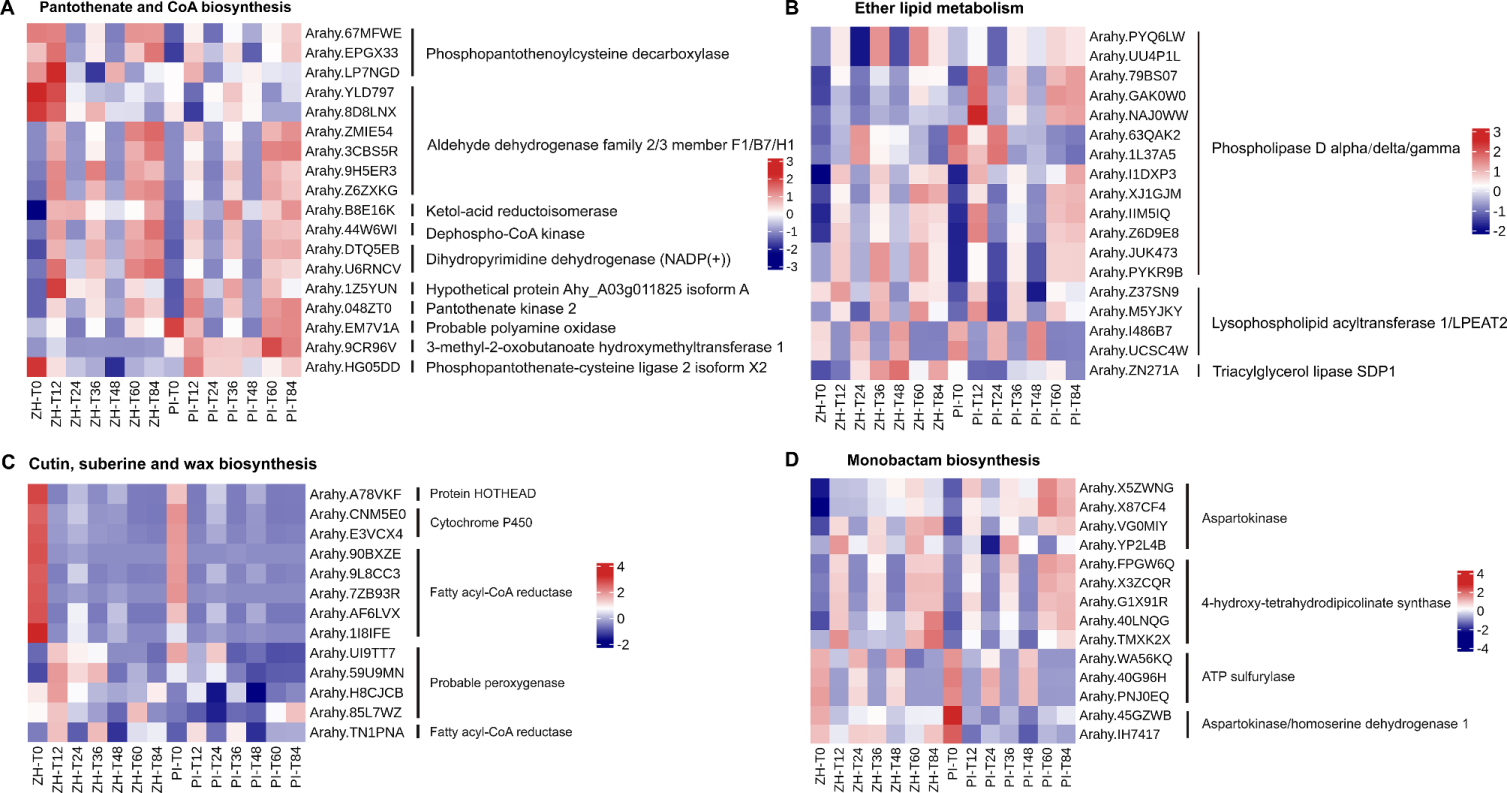
DEGs in the pathways of significant enrichment in resistant variety ZH and susceptible variety PI in the early stage of *D. arachidicola* infection. (**A**) DEGs associated with pantothenate and CoA biosynthesis in ZH and PI at different time-points. (**B**) DEGs associated with ether lipid metabolism in ZH and PI at different time-point. (**C**) DEGs associated with cutin, suberine and wax biosynthesis in ZH and PI at different time-point. (**D**) DEGs associated with monobactam biosynthesis in ZH and PI at different time-point

To identify the genes mediating the web blotch resistance of ZH during the early infection stage, we screened for KEGG pathways that were specifically and significantly enriched among the DEGs in ZH at T12, T24, and T36. Most of the DEGs in the pantothenate and CoA biosynthesis pathway had up-regulated expression levels at T12, with higher expression levels in ZH than in PI. These genes encoded various proteins, including phosphopantothenoylcysteine decarboxylase, aldehyde dehydrogenase family member, ketol-acid reductoisomerase, and dihydropyrimidine dehydrogenase [NADP(+)] (Fig. 4A). Of these genes, the gene *Arahy.9CR96V* encoding 3-methyl-2-oxobutanoate hydroxymethyl transferase 1 was more highly expressed in PI than in ZH. Accordingly, we speculated that this gene may encode a negative regulator of resistance to web blotch disease.

Phospholipase D (PLD) is an important phosphohydrolase involved in many physiological and biochemical processes, including cell lipid metabolism, signal transduction, and biofilm formation. The expression levels of 13 PLD-encoding genes involved in ether lipid metabolism were up-regulated at T12, among which *Arahy.63QAK2* and *Arahy.1L37A5* were up-regulated by 10.6- and 2.5-fold in ZH, but only by 0.6- and 0.8-fold in PI (Fig. 4B).

Only the DEGs of ZH at T12 were significantly enriched to cutin, suberine and wax biosynthesis, but not in PI or at the other time-points. The DEGs in this pathway at T12 encoded protein HOTHEAD, cytochrome P450, fatty acyl-CoA reductase, and peroxygenase. The expression levels of the HOTHEAD gene (*Arahy.A78VKF*) and cytochrome P450 genes (*Arahy.CNM5E0* and *Arahy.E3VCX4*) were down-regulated in ZH and PI at T12, while the expression levels of the peroxygenase genes (*Arahy.UI9TT7*, *Arahy.59U9MN*, and *Arahy.85L7WZ*) were up-regulated in ZH and down-regulated in PI at T12 (Fig. 4C).

Monobactam is a widely applied antibiotic effective against bacteria. Monobactam biosynthesis was a significantly enriched pathway at T24 in ZH. The DEGs involved in this pathway encoded aspartokinase, 4-hydroxy-tetrahydrodipicolinate synthase, and 4-hydroxy-tetrahydrodipicolinate reductase (Fig. 4D), which are rate-limiting enzymes for aspartic acid synthesis. We speculated that the biosynthesis of monobactam and aspartic acid may influence web blotch resistance.

### DEGs associated with HR during the YY187 infection

In plants, HR is a typical resistance-related response involving rapid cell necrosis following the recognition of the non-toxic gene product secreted by the pathogen after the plant-pathogen incompatibility interaction by the protein encoded by a plant resistance (*R*) gene, ultimately leading to ETI. Porphyrin metabolism as well as taurine and hypotaurine metabolism were significantly enriched KEGG pathways only at T60 in ZH, suggesting these pathways may be involved in inducing HR and conferring resistance to YY187 in ZH.

**Fig. 5:**
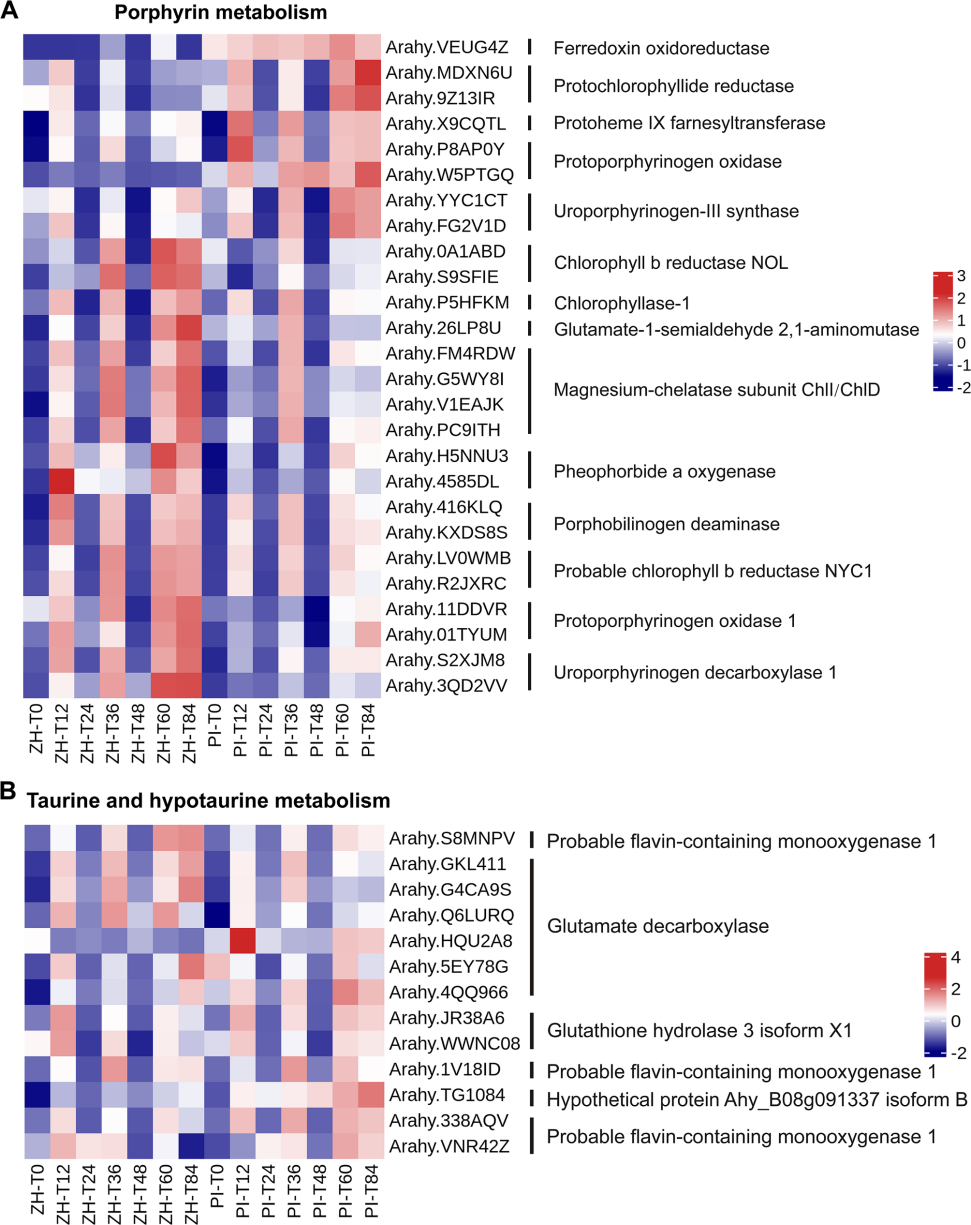
DEGs in the pathways of significant enrichment in resistant variety ZH and susceptible variety PI in the HR stage of *D. arachidicola* infection. (**A**) DEGs associated with porphyrin metabolism in ZH and PI at different time-point. (**B**) DEGs associated with taurine and hypotaurine metabolism in ZH and PI at different time-point

Porphyrin metabolism was an enriched KEGG pathway associated with the DEGs encoding important enzymes involved in degrading excessive amounts of chlorophyll b, namely the chlorophyll(ide) b reductase NYC1, chlorophyllase-1, and pheophorbide an oxygenase (Fig. 5A). Accordingly, these genes may be involved in the induction of HR. In contrast, the genes encoding key enzymes related to chloroplast synthesis, including the chlorophyll(ide) b reductase NOL, magnesium-chelatase subunit ChlD/CHII, porphobilinogen deaminase, protoporphyrinogen oxidase 1, and uroporphyrinogen decarboxylase 1 (Fig. 5A), may be important for increasing web blotch resistance and delaying leaf senescence in ZH.

Taurine and hypotaurine are scavengers of free radicals (i.e., antioxidants) that also maintain calcium homeostasis inside and outside cells and protect the cell membrane from damage. Taurine and hypotaurine metabolism was a significantly enriched pathway at T60 in ZH, indicative of a strong correlation with HR induction and web blotch resistance. The flavin-containing monooxygenase encoded by the DEG associated with this pathway (Fig. 5B) is the rate-limiting enzyme in the tryptophan-dependent pathway mediating indole-3-acetic acid (IAA) biosynthesis. A previous study clarified the relationship between IAA and plant disease resistance. This further illustrates the importance of taurine and hypotaurine metabolic pathways for the resistance of ZH to web blotch.

### DEGs involved in the innate immunity and acquired immunity after the YY187 infection

In this study, HR was observed in both the resistant and susceptible materials at 48 hpi, suggesting that this time-point may separate the innate and acquired immune responses of peanut leaves to YY187. In ZH, cysteine and methionine metabolism was a significantly enriched KEGG pathway at T12 and T48, whereas thiamine metabolism was a significantly enriched KEGG pathway at T36 and T60. Additionally, arginine and proline metabolism was an enriched pathway at T36 and T84 in ZH (Fig. 3), suggesting that these pathways may be involved in the innate immunity and acquired immunity in response to the YY187 infection.

**Fig. 6:**
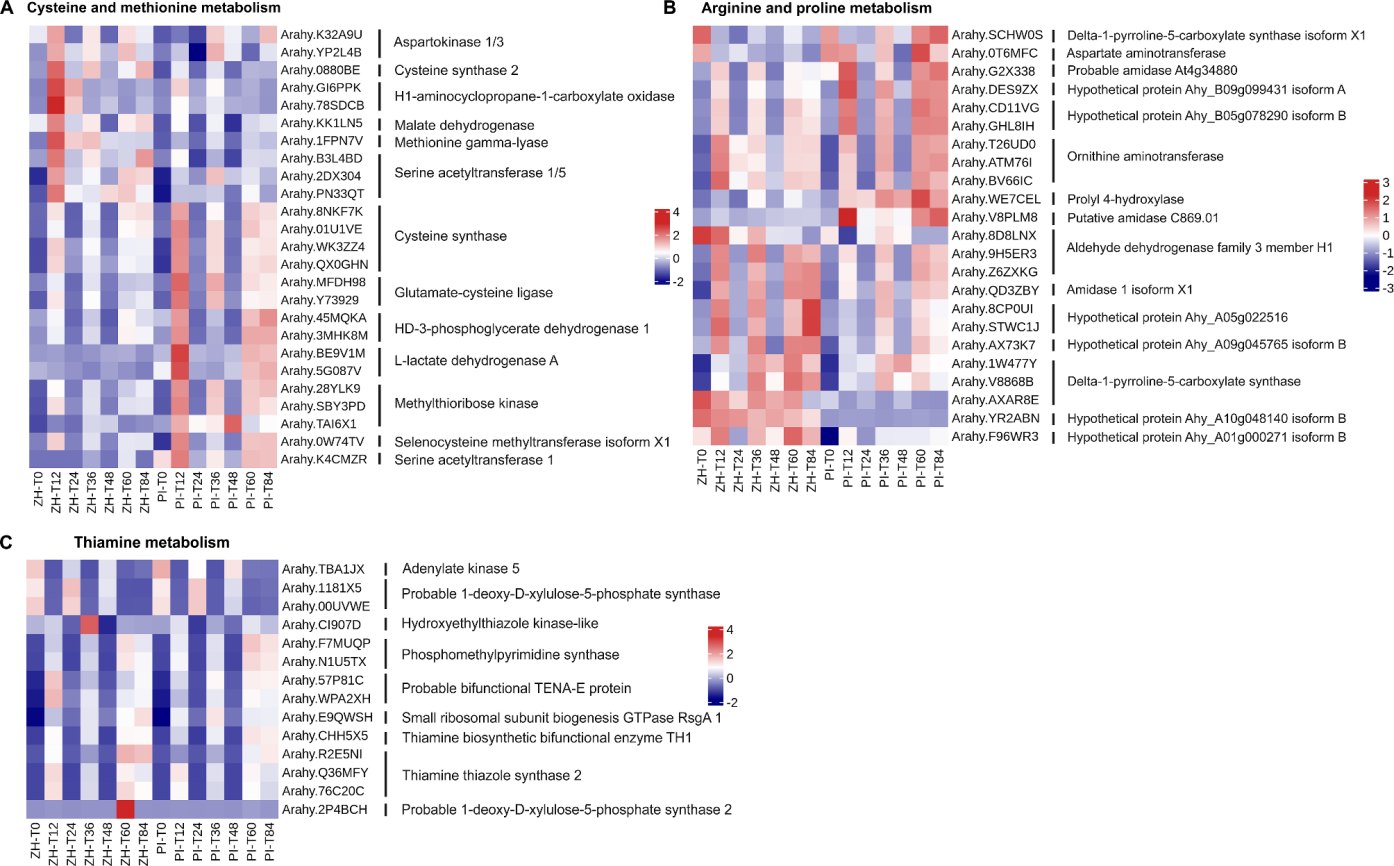
DEGs in the pathways of significant enrichment in resistant variety ZH and susceptible variety PI in the early and HR stage of *D. arachidicola* infection. (**A**) DEGs associated with cysteine and methionine metabolism in ZH and PI at different time-point. (**B**) DEGs associated with arginine and proline metabolism in ZH and PI at different time-point. (**C**) DEGs associated with thiamine metabolism in ZH and PI at different time-point

In the cysteine and methionine metabolism KEGG pathway, 40% of the DEGs had significantly up-regulated expression levels in ZH, and their expression was significantly induced at T12, while the expression levels of the genes encoding aspartate kinase, cysteine synthetase, and serine acetyltransferase were up-regulated at T36, T60, and T84; these enzymes may be related to the differential disease resistance between ZH and PI (Fig. 6A). In addition, the expression levels of 60% of the DEGs were significantly up-regulated in PI, and their expression was significantly induced at T12 and then significantly up-regulated at T36, T60, and T84; these genes may be negatively correlated with peanut resistance to web blotch (Fig. 6A). In the arginine and proline metabolism KEGG pathway, the expression levels of approximately 50% of the DEGs were up-regulated in ZH and PI, of which the expression of *Arahy.WE7CEL*, which was significantly up-regulated in PI infected with YY187, was not significantly induced in ZH, suggesting that the gene may encode a negative regulator of web blotch resistance (Fig. 6B). Among the DEGs in the thiamine metabolism KEGG pathway, the expression of *Arahy.CI907D* was significantly up-regulated only at T36 in ZH, while the expression of *Arahy.2P4BCH* was significantly up-regulated only at T60 in ZH (Fig. 6C). The thiamine metabolism was significantly enriched only among the DEGs at T36 and T60 in ZH. Hence, these two genes may be crucial for web blotch resistance.

### Analysis of the protein–protein interaction network constructed for the genes in the enriched pathways in ZH

**Fig. 7:**
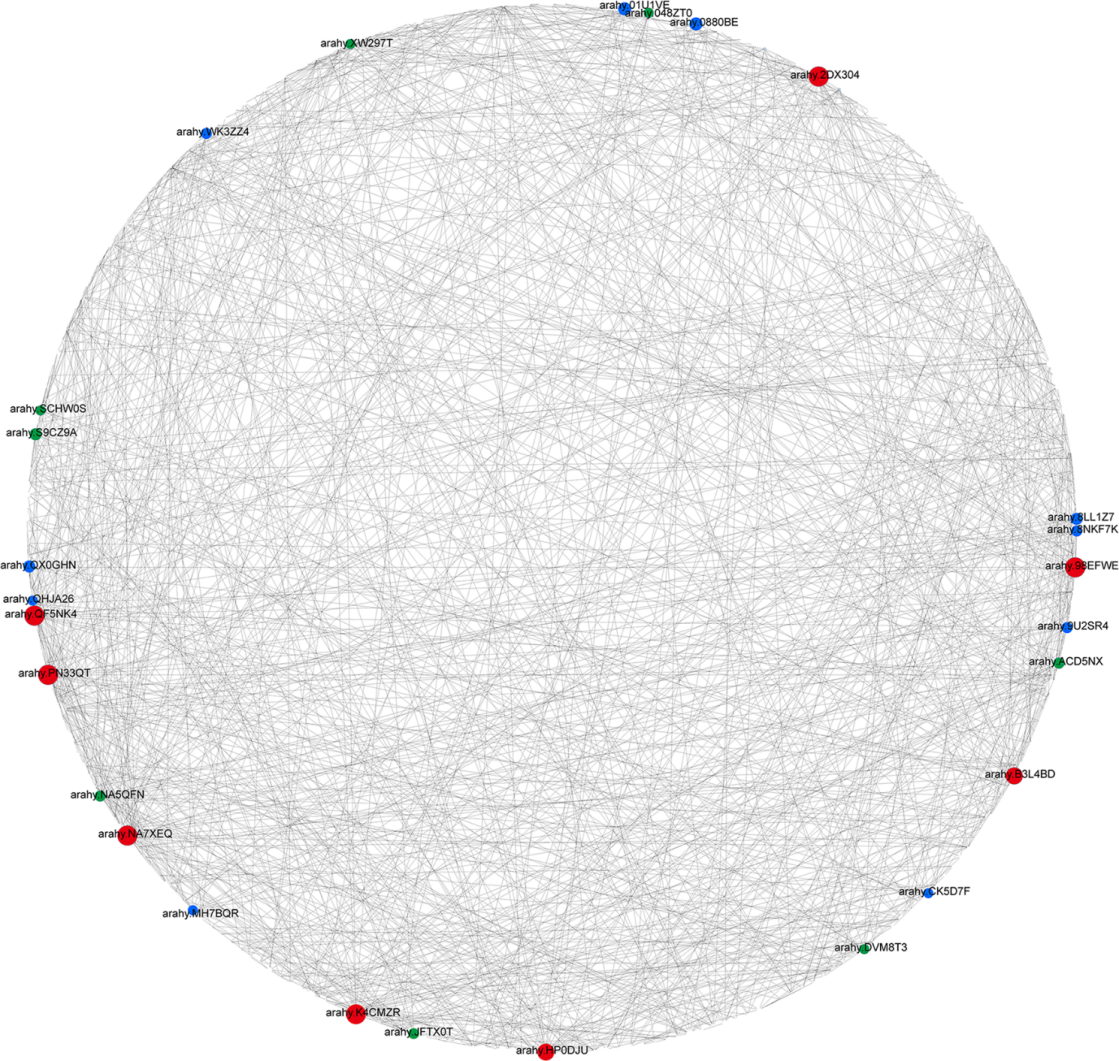
Protein-protein interaction (PPI) network for the genes in the specifically enriched pathways in ZH. Hub genes are shown as red nodes (serine acetyltransferase), blue nodes (cysteine synthase), and green nodes (other)

To analyze the hub genes related to web blotch resistance in the enriched pathways, we performed a protein–protein interaction (PPI) network analysis involving all of the genes associated with the enriched pathways in ZH. The PPI network analysis revealed 26 hub genes that may be important for the resistance to YY187 (Fig. 7). sixteen of these genes encode serine acetyltransferases (red circle) and cysteine synthases (blue circle), respectively (Fig. 7; Table 1). Although both enzymes are important for the synthesis of cysteine, serine acetyltransferase is the rate-limiting enzyme. Hence, cysteine and methionine metabolism may be a vital pathway mediating the resistance of ZH to YY187. Furthermore, the antioxidant activity of cysteine may be a critical factor affecting the resistance of peanut to pathogens.

**Table 1:** Functional annotation of the candidate genes

Number	Gene Name	Functional annotation
1	*Arahy.2DX304*	Serine acetyltransferase
2	*Arahy.98EFWE*	
3	*Arahy.B3L4BD*	
4	*Arahy.HP0DJU*	
5	*Arahy.K4CMZR*	
6	*Arahy.NA7XEQ*	
7	*Arahy.PN33QT*	
8	*Arahy.QF5NK4*	
9	*Arahy.01U1VE*	Cysteine synthase
10	*Arahy.0880BE*	
11	*Arahy.8LL1Z7*	
12	*Arahy.8NKF7K*	
13	*Arahy.9U2SR4*	
14	*Arahy.CK5D7F*	
15	*Arahy.QX0GHN*	
16	*Arahy.WK3ZZ4*	
17	*Arahy.MH7BQR*	Bifunctional L-3-cyanoalanine synthase/cysteine synthase
18	*Arahy.QHJA26*	
19	*Arahy.DVM8T3*	Oxygen-dependent coproporphyrinogen-III oxidase
20	*Arahy.S9CZ9A*	
21	*Arahy.ACD5NX*	Pantothenate kinase
22	*Arahy.048ZT0*	
23	*Arahy.JFTX0T*	
24	*Arahy.XW297T*	Phosphoserine aminotransferase
25	*Arahy.NA5QFN*	Delta-1-pyrroline-5-carboxylate synthase isoform X1
26	*Arahy.SCHW0S*	

### Validation of the RNA‑seq data via a qRT‑PCR analysis

**Fig. 8:**
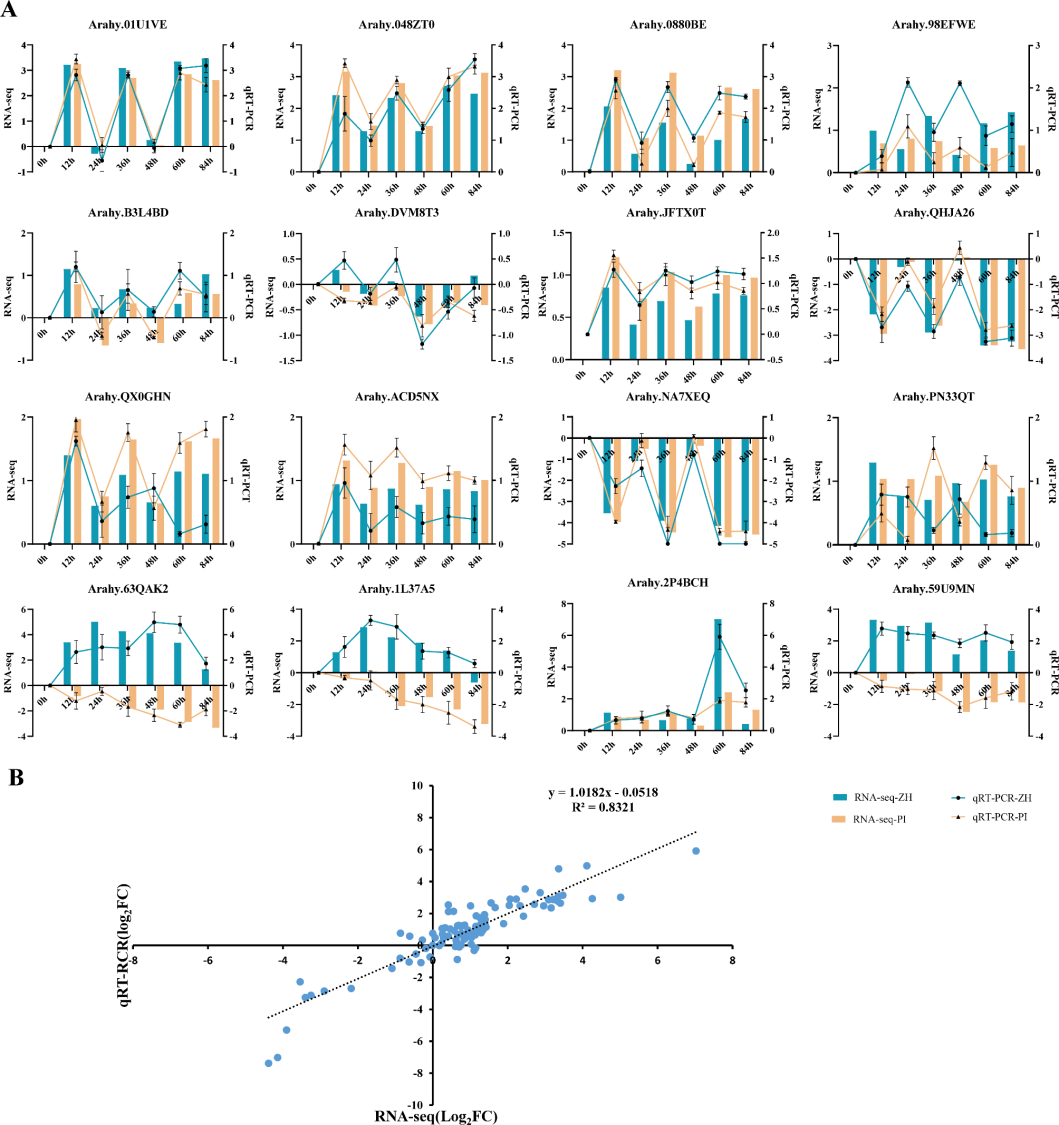
Validation of RNA-seq data by RT-qPCR. (**A**) qRT-PCR verified that 16 genes related to the resistance to web blotch. The line chart shows the relative expression measured by qRT-PCR, and the bar chart shows the expression level (log_2_FC) measured by RNA-seq. The log_2_FC of the transcript levels in the inoculated samples compared to T0 is shown. The error bar indicates that the standard error comes from three independent organisms and three technical repetitions of qRT-PCR assays. (**B**) Correlation of transcript expression between qRT-PCR and RNA-Seq results

To verify the genes identified in this study that may be involved in the defense of peanut web blotch disease, we have verified the expression levels of 16 candidate genes in ZH and PI by conducting a quantitative real-time PCR (qRT-PCR) analysis (Table S2) (Fig. 8A). The relative expression values of all tested gene were calculated by using the *Actin* gene which was composed of expression. The correlation between qRT-PCR and RNA-seq expression values was analyzed. The results showed that the expression profiles of these genes verified by qRT-PCR was in line with RNA-Seq data (R^2^ = 0.8321) (Fig. 8B).

## Discussion

To systematically investigate the molecular basis of the resistance of peanut to *D. arachidicola* and thoroughly clarify the pathogenesis of web blotch, we studied the *D. arachidicola* infection characteristics on peanut varieties that differed in terms of resistance. We also conducted a time-course RNA-seq analysis using the leaves collected from seedlings that differed regarding resistance to reveal the transcriptional changes associated with a *D. arachidicola* infection.

We compared the pathogen infection characteristics on the leaves of ZH (resistant) and PI (susceptible) at seven time-points (0, 12, 24, 36, 48, 60, and 84 hpi), which coincided with different pathogen infection stages on the leaves. Conidia started to germinate at 12 hpi, appressoria were generated at 24 hpi, papillae began to appear at 36 hpi, and HR was detectable at 48 hpi. The formation of papillae, which was first reported by deBary in 1863, is one of the earliest observed plant defense responses that inhibit fungal infections [27]. Additionally, HR, which is probably the most well-studied plant response to pathogen infections, involves rapid localized cell death induced by the recognition of pathogens by the R protein, thereby limiting the pathogen to the infection site and leading to resistance [28, 29]. The papilla formation rate (36 hpi) and HR rate (60 and 84 hpi) were significantly higher for ZH than PI (Fig. 1), indicating that the number of papillae and the extent of HR at the infection site are important factors associated with web blotch resistance. These results are consistent with those of previous studies that determined the presence of papillae indicates PTI has been activated (first layer of defense) and HR indicates that ETI has been activated (second layer of defense) [30]. In the current study, 48 hpi seemed to be the dividing line between PTI and ETI.

To gain insights into the molecular basis of peanut resistance to *D. arachidicola*, an RNA-seq analysis was performed. The process of pathogen infection was divided into different time periods, and observed by microscope. According to the analysis of differential gene expression, there were more DEGs at 12, 36, 60, and 84 hpi than at the other time-points, which corresponded to the spore germination stage (T12), papilla production stage (T36), and HR stage (T60 and T84), respectively. T12 is the spore germination period, and it is also the time when plants recognize conservative pathogen/microorganism-related molecular patterns (P/MAMP) through PRRs on the cell surface and activate the first defense system of plants. T36 is the period of producing physical barrier papilla structure, and T60 is the period of stably producing HR. The stable appearance of HR represents the role of resistance gene represented by leucine-rich repetitive sequence (NB-LRR), and also means pathogen specificity. Therefore, a large number of DEGs appeared in these periods (Fig. 2), indicating that the immune response in these periods was stronger than that in T24 and T48. In fact, the interaction between pathogens and plant cells is like an arms race. When plants are infected by pathogens, they will immediately stimulate an immune response to prevent infection. Pathogens will also crack the immune response of plants by various means, such as by inhibiting the expression of related genes. This may also be the reason for the small number of deg in T24 and T48. In addition, there were more up-regulated DEGs than down-regulated DEGs at all time-points, which is in accordance with the findings of an earlier study on the infection of peanut by *Aspergillus flavus* [31]. During the peanut–pathogen interaction, the expression of many peanut genes is induced in response to the pathogen. Moreover, after the *D. arachidicola* infection, there were more DEGs in PI (susceptible) than in ZH (resistant). Earlier research on the effects of *Magnaporthe oryzae* on rice plants resistant and susceptible to blast detected more DEGs in the susceptible rice than in the resistant rice [32]. So, the successful infection of susceptible varieties by pathogens has led to the development of complex adaptive mechanisms that increase the chances of survival [33].

The plant leaf surface is covered by a hydrophobic epidermal wax that minimizes the adhesion of water and other particles, including potentially invasive microorganisms. The ability of a fungal pathogen to penetrate plants is a major determinant of a successful infection. The waxy layer on the plant cell wall and leaf surface represents the first line of defense against pathogens [34]. During spore germination, many powdery mildew fungi secrete lyases, such as lipases, esterases, and keratinases, that can degrade the outermost barrier of plant cells [35]. The cutin, suberine and wax biosynthesis pathway was significantly enriched among the DEGs at 12 hpi in ZH (Fig. 3), indicating peanut resistance to web blotch may involve the strengthening of the protective outer surface. An earlier comparison of the development of fungi on leaves and artificial surfaces showed that the wax on leaves can hinder appressorium formation because of its hydrophobicity [36]. Alternatively, the wax prevents the diffusion of chemical signals from the cuticle, which is necessary for fungal development [37]. In addition to the plant cell wall, the first line of defense also includes antibacterial enzymes and secondary metabolites [10]. This was also demonstrated by the significantly enriched monobactam biosynthesis pathway among the DEGs at 24 hpi in ZH (Fig. 3). Although monobactam is a monocyclic beta-lactam antibiotic widely used against bacteria, the enzymes encoded by the DEGs in this pathway (i.e., aspartate kinase and 4-hydroxy-tetrahydropyridine dicarboxylate synthase) (Fig. 4D) are also rate-limiting enzymes during aspartate synthesis [38] and lysine synthesis [39]. Monobactam biosynthesis combined with the production of aspartic acid and lysine may be involved in the mechanism underlying the resistance to web blotch. The second physical barrier that prevents pathogen invasions is the cell membrane. Ether lipid is an important cell membrane component that affects a variety of biological functions [40]. The ether lipid metabolism KEGG pathway was significantly enriched among the DEGs at 24 hpi in ZH. In addition, 72% of the DEGs in this pathway encoded PLD (Fig. 4B), which is an enzyme that catalyzes a reaction during the initial stage of lipid hydrolysis. Phospholipase D (PLD) is an enzyme belonging to the PLD superfamily that catalyzes the hydrolysis of phospholipids by phosphodiester bonds, and some hydroxy-containing compounds to generate phospholipic acid (PA) and various head groups, such as choline or ethanolamine [41]. At present, a large number of reports have confirmed the role of PLD in plant defense. PLDβ1t regulates actin cytoskeleton dynamics or bridges the interface between cell membrane and cytoskeleton through its interaction with actin, thus participating in plant defense response to pathogen attack [42]. PLD responds to the perception of PAMPs and the disturbance of ETI during PTI by regulating the changes of cytoskeleton array and the abundance of actin [43, 44]. 13 PLD-encoding genes were enriched in the pathway of class B lipid metabolism which was significantly enriched in the early stage of infection (Fig. 4B). These genes are mainly up-regulated in T12, T36, T60 and T84 of ZH and PI. The previous cytological examination and analysis confirmed the interaction state between pathogens and plant cells in different periods, so we can speculate that the genes up-regulated at T12 and T36 may participate in the interaction between pathogens and plants by adjusting the cytoskeleton or PAMP perception in the cell membrane, while the genes up-regulated at T60 and T84 may participate in the interaction between pathogens and plants by participating in the HR response induced by the oxygen explosion of ROS.

Thiamine is a B complex vitamin that is produced by plants and microorganisms. Studies have shown that a thiamine treatment has a significant inhibitory effect on rice sheath blight [45], soybean carbon rot [46], tobacco black shank [47], and grape pulp mold [48]. Specifically, fungal growth is reportedly limited to the infection site, which may be because thiamine, as a defense activator, induces rapid opening of defense response [49]. Thiamine-induced defense reactions include the production of H_2_O_2_, the synthesis of callose, the expression of defense-related genes, the accumulation of phenols, and the death of HR-like cells [47, 49]. In the current study, the thiamine metabolism KEGG pathway was enriched among the DEGs at 36 and 60 hpi in ZH (Fig. 3), suggestive of the correlation between thiamine and papillae (callose) and HR in the web blotch resistance mechanism of ZH. Similarly, the arginine and proline metabolism KEGG pathway is also associated with the disease resistance related to papillae and HR. The arginine and proline metabolism pathway was enriched among the DEGs at 36 and 84 hpi in ZH (Fig. 3). In plants, arginine is the main stored and transported forms of organic nitrogen, while also serving as the precursor of polyamines and NO, which are important for the distribution and circulation of nitrogen in plants [50, 51]. Polyamines and NO are critical for plant stress and disease resistance. During a tobacco mosaic virus infection, polyamines induce the production of H_2_O_2_, which leads to HR-like cell death, while polyamine biosynthesis inhibitors (α-difluoromethyl-ornithine) restrict the accumulation of polyamines and decrease the HR rate [52]. As early as 1998, some scholars proposed that NO functions as a signaling molecule influencing plant disease resistance [16]. The relative levels of NO and H_2_O_2_ can mediate HR-related cell death in soybean cells [53]. In tobacco cells, increases in NO and H_2_O_2_ contents lead to typical programmed cell death and related biochemical changes [54]. Proline is a protein-derived amino acid that accumulates as a beneficial solute in plants under stress and non-stress conditions. In plants, proline metabolism protects against stress by maintaining the NADPH/NADP^+^ balance and GSH level, while also promoting the oxygen explosion during a pathogen infection [55–57].

Porphyrins are the basis of many important plant pigments, including chlorophyll, heme, and carotenoids. Their metabolism is crucial for plant growth and development as well as photosynthesis. The overexpression of the gene encoding human protoporphyrinogen IX oxidase in rice results in a considerable increase in the leaf porphyrin content (relative to the wild-type level), ultimately leading to the accumulation of large amounts of ROS [58]. A mutation to the coproporphyrinogen III oxidase-encoding gene in the porphyrin pathway promotes the degradation of chlorophyll and the accumulation of ROS in maize leaves, resulting in yellow-green necrosis [59]. In the present study, the porphyrin metabolism KEGG pathway was specifically enriched among the DEGs at 60 hpi in ZH (Fig. 3). Additionally, the uroporphyrinogen decarboxylase-encoding gene in this pathway was expressed at significantly higher levels in ZH than in PI (Fig. 5A). Hence, this pathway may contribute to peanut web blotch resistance through ROS-mediated HR. The taurine and hypotaurine metabolism KEGG pathway was also enriched among the DEGs at 60 hpi in ZH. Studies have shown that hypotaurine can react with the hydroxyl radical (OH) and hypochlorous acid (HOCL), with a reaction rate that is 100- to 10,000-times higher than that of taurine, making it an excellent antioxidant [60, 61]. Although taurine appears to lack antioxidant activity in the cell, hypotaurine can be converted to taurine via an interaction with ROS (superoxide, OH, and H_2_O_2_) [62]. Therefore, taurine and hypotaurine metabolism is likely important for the web blotch resistance of peanut.

The PPI network-based analysis of all of the genes in these pathways significantly enriched at all time-points on ZH revealed that 16 of the 26 candidate genes were associated with cysteine and methionine metabolism, of which eight were serine acetyltransferase genes and eight were cysteine synthase genes (Fig. 7; Table 1). These two enzymes are vital for cysteine synthesis. As a reduced sulfur donor molecule, cysteine is involved in the synthesis of various essential biomolecules and defense compounds and is a critical component of plant metabolism [63]. Cysteine and its derivatives participate in redox processes in various cells (e.g., antioxidant activities of glutathione) [64, 65]. In addition, the sulfhydryl group of cysteine, which is usually located at its active site, is essential for many enzymatic reactions and modulates various defense processes involving enzymes [66]. In tomato, a papain-like cysteine proteinase is necessary for the Cf-2-mediated resistance to *Cladosporium fulvum* and for the inhibition of the self-necrosis of leaf cells [67]. The wheat gene encoding a cysteine-rich receptor-like kinase (TaCRK2) positively regulates the HR induced by a wheat smut infection [68]. In addition to enzymes, cysteine is a part of various other proteins involved in defense responses. For example, cysteine-rich antimicrobial peptides can effectively protect plants from various diseases, including sclerotinia stem rot and black spot [69]. In addition, cysteine homeostasis is also an important factor affecting plant immunity. Knocking out the O-acetylserine (thiol) lyase gene reportedly leads to increased sensitivity to biotrophic and necrotrophic pathogens [70]. In terms of metabolism, L-cysteine is used to produce glutathione (GSH), hydrogen sulfide (H_2_S), cysteine sulfonic acid, taurine, pyruvate, and inorganic sulfur, which function as antioxidants [71]. Thus, the resistance of peanut to web blotch caused by *D. arachidicola* appears to be coordinated through the metabolism of cysteine.

**Fig. 9:**
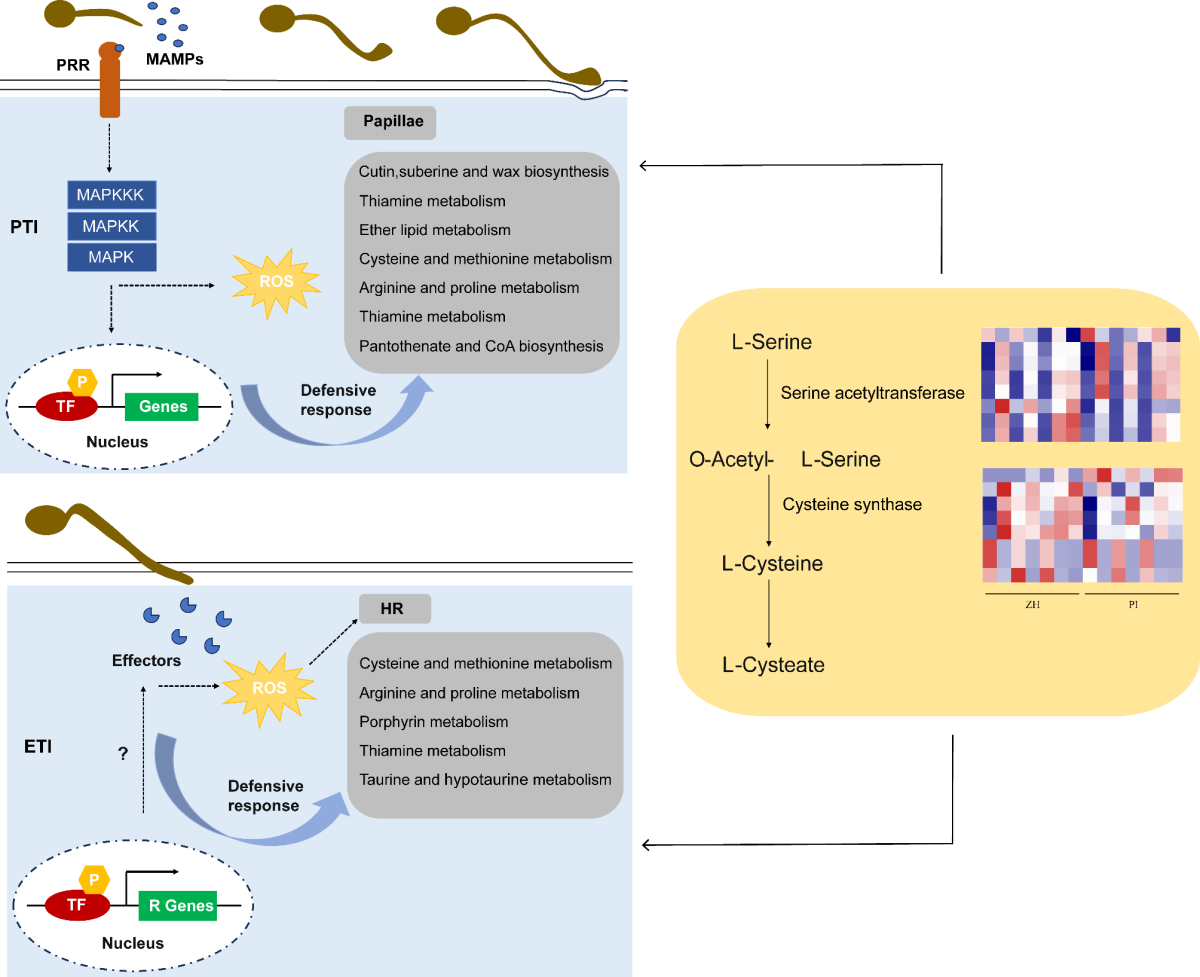
Potential mechanism underlying peanut resistance to *D. arachidicola*

Finally, through the above research and analysis, we seem to have a preliminary understanding of the resistance mechanism of peanut web blotch (Fig. 9). Plants have developed two natural immune systems to defend against pathogens [72–74], and so have peanuts. Primary innate immunity is the first line of defense. MAMPs are recognized by PRRs, and primary defense reactions (PTI) are induced, such as cell wall changes, callose deposition and defense-related proteins (including chitinase, glucanase and protease) accumulation, which have a negative impact on pathogen colonization [75]. Papilla is a common component of PTI reaction [76], which forms a physical barrier at the pathogen detection site and restricts the entry of pathogens. And in this process, many metabolic pathways are involved, such as cutin, suberine and wax biosynthesis, cystine and methionine metabolism, pantothenate and CoA biosynthesis, ether lipid metabolism, etc. (Fig. 9). There are also antibacterial secondary metabolic pathways such as monobactam biosynthesis and thiamine metabolism. These physical barriers and metabolic pathways work together to protect peanuts from *D. arachidicola*. However, potential pathogens will secrete effectors that interact with host targets and inhibit the primary defense response of peanut cells. Therefore, in the secondary immune system, peanut recognizes the effector released by pathogen through resistance protein (RPs), which triggers the RPs-mediated secondary defense response, and finally leads to the appearance of local strong HR, which prevents the growth of *D. arachidicola* [73, 74]. In this process, many metabolic pathways are involved, such as cysteine and methionine metabolism, thiamine metabolism, taurine and hypotaurine metabolism (Fig. 9). Additionally, two important enzymes in cysteine metabolism pathway, serine acetyltransferase and cysteine synthetase, may play a key role in the whole disease resistance process.

## Conclusion

In this study, the mechanism underlying the resistance of peanut to web blotch was explored by analyzing peanut leaf cytological processes and structures following an infection by *D. arachidicola* and by comparing the transcriptomes of ZH (resistant) and PI (susceptible). We observed that the papilla structure and HR produced at the early stage of infection may help to explain the web blotch resistance of ZH. The significantly enriched KEGG metabolic pathways in ZH during different infection stages were identified, including cutin, suberine and wax biosynthesis, monobactam biosynthesis, ether lipid metabolism, porphyrin metabolism, taurine and hypotaurine metabolism, and cysteine and methionine metabolism. Thus, these pathways also likely contribute to the resistance of ZH to web blotch. According to the constructed PPI network, most of the candidate genes encode enzymes in the cysteine synthesis pathway, indicative of the importance of cysteine and methionine metabolism throughout the *D. arachidicola* infection process (Fig. 9). The study findings provide an important theoretical basis for future investigations on the molecular mechanism mediating peanut resistance to web blotch and for identifying the genes responsible for the resistance.

## Materials and methods

### Cultivation and collection of pathogen spores

The pathogen used in this study was *D. arachidicola* strain YY187, which was obtained from symptomatic peanut leaves collected in Yuanyang, Henan, China via single spore isolation and then preserved by the Crop Protection Laboratory of the Henan Academy of Agricultural Sciences [77]. This strain is highly virulent, and the genome sequencing has been completed [77]. The spores were propagated on oatmeal agar medium, with new conidia obtained after a 20-day incubation at 25 ± 2 °C in darkness. The oat agar (OA) mediums containing pycnidiums were ground with sterile water (1:1, OA medium : sterile water, v/v) to release spores, and then the spore-containing suspension was collected by filtration with 4 layers of gauze. The concentration of the spore suspension was observed and calculated with a blood cell counting plate.

### Cultivation of plants, inoculation, and collection of leaves

Resistant variety Zheng8903 (ZH) and susceptible variety PI343384 (PI) were grown in a growth chamber at 25 °C with a 14-h light (8,000 lx)/10-h dark cycle and 60–90% humidity at the Henan Academy of Agricultural Sciences. When seedlings reached the 6- to 8-leaf stage, they were sprayed with a YY187 spore suspension (2 × 10^6^ conidia/ml) supplemented with 0.1% Tween 20 [78], after which the parietal leaves were labeled with a string. Next, the relative humidity in the growth chamber was adjusted to 85%, but the other conditions were unchanged.

### Leaf staining and cytological observation

For the cytological examination of the YY187 infection in ZH and PI, a histopathological analysis was performed using the leaves collected at 0, 12, 24, 36, 48, 60, and 84 hpi. At each time-point, two leaflets of a compound leaf were obtained from three plants for a total of six leaf samples. The veins of each blade were removed using a scalpel and divided into two segments. The leaves were soaked in a DAB (1 mg/ml, pH 3.8) solution for 24 h and decolorized using an acetic acid:ethanol (1:3, v/v) solution. The leaves were rinsed with distilled water 2–3 times, soaked in trypan blue dye, placed in boiling water for 5 min, rinsed with sterile water 2–3 times, and placed in saturated chloral hydrate until they became translucent. They were then rinsed with 20% glycerin and leaf specimens were prepared. All leaves were examined using the DM2500 phase contrast microscope (Leica Microsystems GmbH, Germany). For the leaf samples collected at 0, 12, 24, 36, and 48 hpi, 300 conidia were detected in 12 leaf segments. The confirmed spore germination rate and appressorium formation rate were calculated. A total of 100 infection sites on 12 leaf segments were examined to determine the papilla generation rate. For the leaf samples collected at 48, 60, and 84 hpi, 100 infection sites on 12 leaf segments were analyzed to clarify the formation rate of HR.

### Transcriptomic analysis of ZH and PI in response to the YY187 Infection

For the transcriptomic analysis, leaf samples were collected from ZH and PI plants at 0, 12, 24, 36, 48, 60, and 84 hpi. Seven time points, three biological repeats at each time point, a total of 42 samples. Total RNA was extracted from each leaf sample using the RNAprep Pure Plant Kit (Tiangen) and then used to construct cDNA libraries for the RNA-seq analysis completed using the Illumina high-throughput sequencing platform (Gene Denovo, Guangzhou, China). After removing the low-quality reads (Fastp 0.18.0), the clean reads were aligned with the peanut reference genome (https://peanutbase.org/data/v2/Arachis/hypogaea/genomes/Tifrunner.gnm2.J5K5/) using HISAT2 (v2.2.4) [79] with ‘-rna-strandness RF’ and other parameters set as a default. The mapped reads of each sample were assembled by using StringTie (v1.3.1) [80, 81] in a reference-based approach. For each transcription region, a FPKM (fragment per kilobase of transcript per million mapped reads) value was calculated to quantify its expression abundance and variations, using RSEM (v1.2.12) [82] software. Differential gene expression was calculated using the R package DESeq (v1.10.1) while considering the independent biological sample triplicates. We used |log_2_(fold-change)|≥2 and FDR<0.01 as the criteria to identify the DEGs in ZH and PI. The RNA-seq raw data were deposited in the NCBI SRA database (accession number: PRJNA983918).

Gene Ontology (GO) [83] enrichment analysis and Kyoto Encyclopedia of Genes and Genomes (KEGG) [84, 85] pathway analysis of the DEGs were performed using the GSEA (v4.1.0) software [86]; FDR (q value) < 0.05 was set as the threshold for identifying significantly enriched GO terms and KEGG pathways.

Protein-protein interaction network was identified using String v10 [87], which determined genes as nodes and interaction as lines in a network. The network file was visualized using Cytoscape (v3.7.1) [88] software to present a core and hub gene biological interact.

### Quantitative real-time PCR analysis

To ensure consistency, the RNA used for the qRT-PCR analysis was the same as that used for the RNA-seq analysis. The extracted total RNA and the PrimeScript™ II 1st Strand cDNA Synthesis Kit (Takara, Dalian, China) were used to synthesize cDNA. The qRT-PCR analysis was performed using the PowerUp SYBR Master Mix (Applied Biosystems, CA, USA) and the QuantStudio 5 Real-time PCR system (Applied Biosystems). The PCR program was as follows: 95 °C for 2 min; 40 cycles of 95 °C for 15 s and 60 °C for 1 min. The analysis was completed using three biological replicates, each comprising three technical replicates. Gene expression levels were determined according to the 2^−ΔΔCt^ method [89], with standard errors calculated for the three biological replicates. A peanut housekeeping *Actin* was selected as the internal reference control.

### Statistical analysis

Data were calculated and analyzed using the GraphPad Prism (v.8.3.0) software (GraphPad Software Inc., La Jolla, CA, USA). A one-way ANOVA was used to determine significant differences and Student’s *t-*test was used to evaluate the statistical significance of the qRT-PCR results (*P < 0.05; **P < 0.01). FPKM values of all genes are shown in Table S3.

### Electronic supplementary material

Below is the link to the electronic supplementary material.

Supplementary Material 1

Supplementary Material 2

## Data Availability

Raw data of the RNA-sequencing experiment supporting this publication were deposited in the National Center for Biotechnology Information (NCBI) and be accessed in the sequence read archive (SRA) database (https://www.ncbi.nlm.nih.gov/sra). The accession number is PRJNA983918 (https://www.ncbi.nlm.nih.gov/bioproject/PRJNA983918).
