# Supplementary material for: Cytological and transcriptomic analysis to unveil the mechanism of web blotch resistance in Peanut
Source: BMC Plant Biol. 2023 Oct 26;23:518. doi: 10.1186/s12870-023-04545-9 (PMC10601179; doi:10.1186/s12870-023-04545-9)
Supplement: Supplementary file 2 — Supplementary Material 2 [file 12870_2023_4545_MOESM2_ESM.pdf]

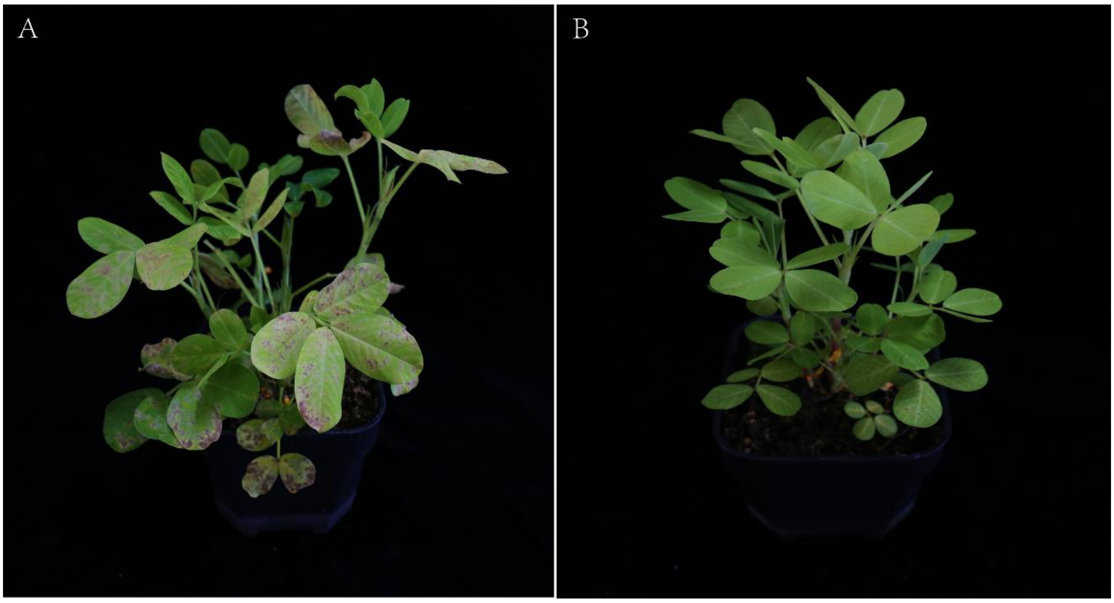

Fig. S1 Phenotypes of the susceptible variety PI (A) and the resistant variety ZH (B) inoculated with YY187 at 14dpi.

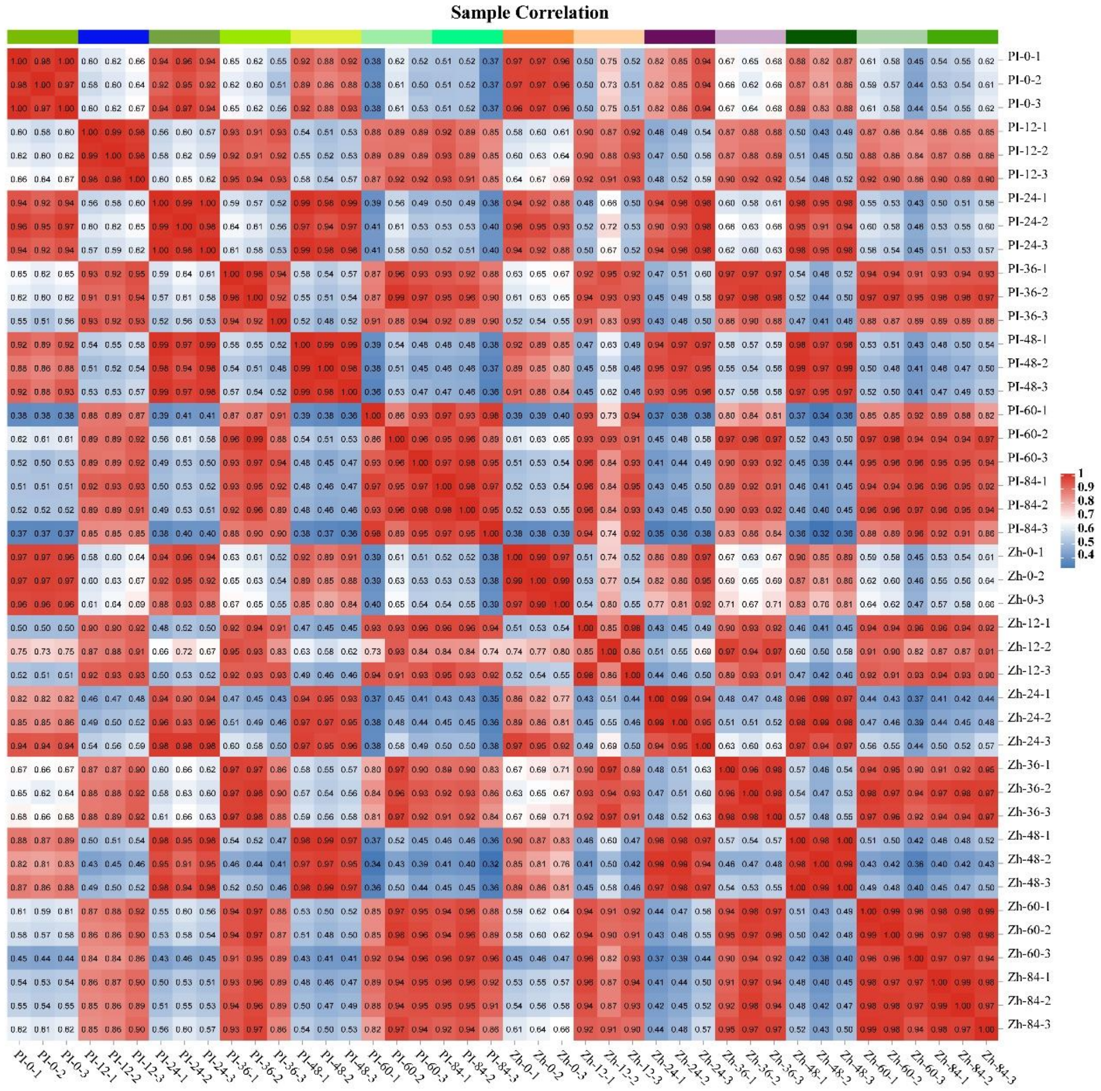

Fig. S2 Pairwise Pearson's correlation coefficients of the sequencing data of 42 samples.

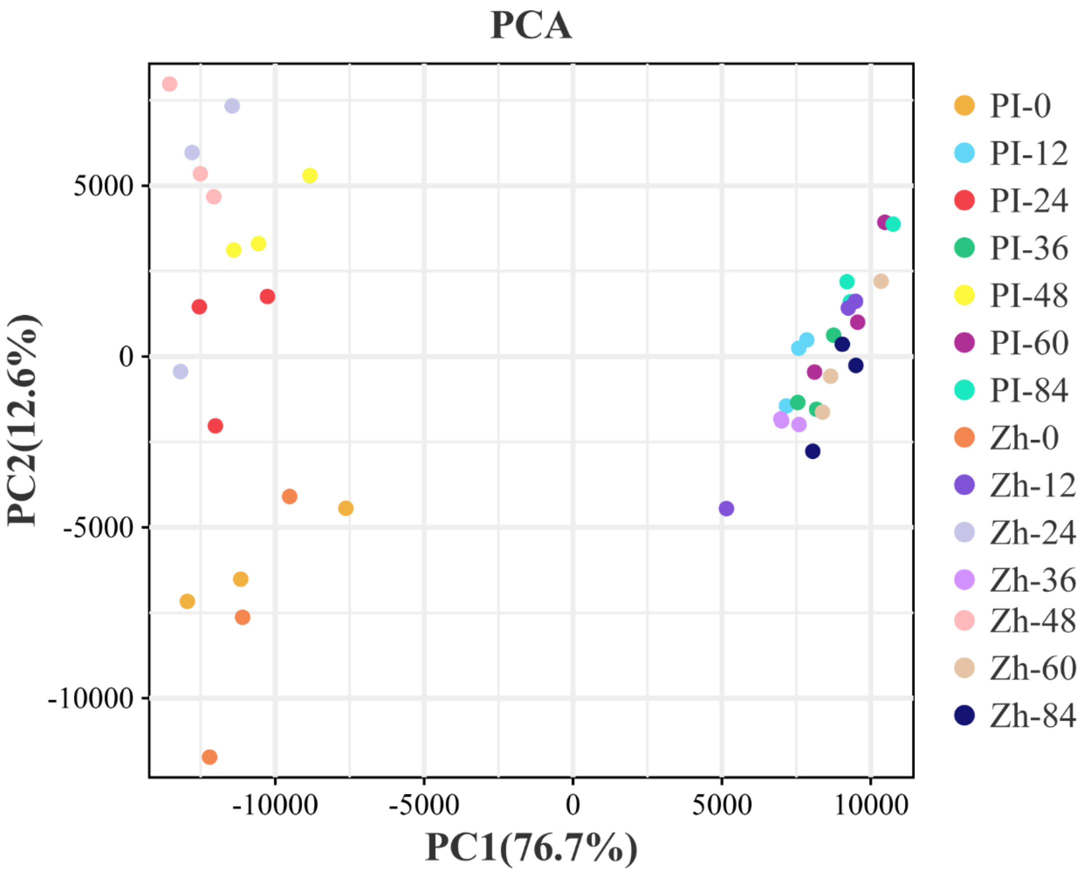

Fig. S3 Principal component analysis of the sequencing data of 42 samples.

A

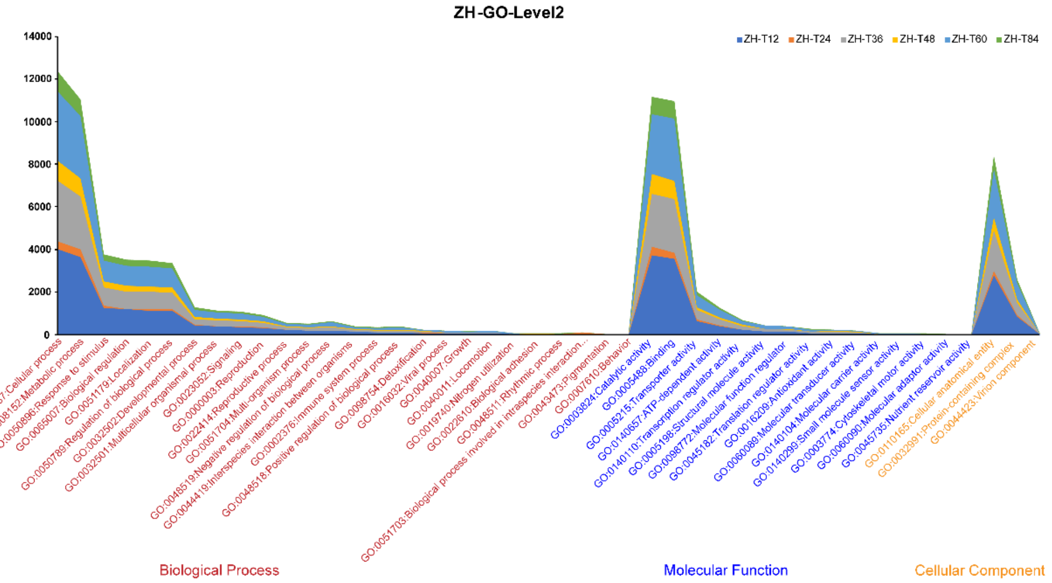

B

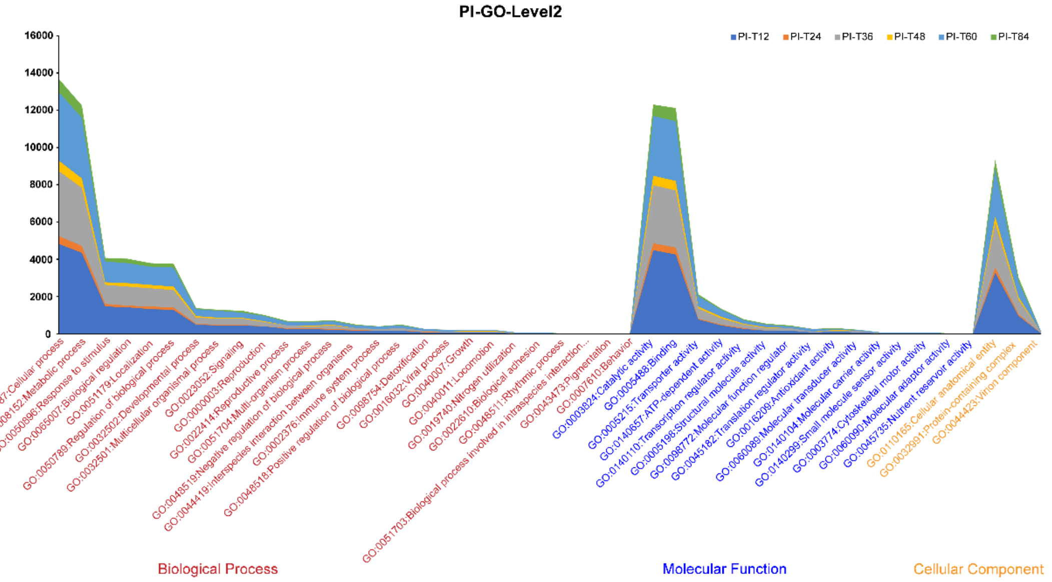

Fig. S4 The level2 of GO enrichment analysis of uniquely DEGs in ZH(A) and PI(B) at T12, T24, T36, T48, T60, and T84.
